# Decision regret after prostate biopsy for prostate cancer diagnosis: a Korean multicenter cohort study

**DOI:** 10.1186/s12889-024-19179-1

**Published:** 2024-06-28

**Authors:** Hwanik Kim, Woojin Bang, Myungsun Shim, Cheol Young Oh, Sung Yong Cho, Mun Su Chung, Dae Sung Cho, Sun Il Kim, Seung Hwan Lee, Kyo Chul Koo, Kwang Suk Lee, Jin Seon Cho

**Affiliations:** 1 Department of Urology, Hallym University Sacred Heart Hospital, Hallym University College of Medicine, Chuncheon, Korea; 2 Department of Urology, Inje University Ilsan Paik Hospital, Inje University College of Medicine, Goyang, Korea; 3 Department of Urology, International St. Mary’s Hospital, Catholic Kwandong University, Incheon, Korea; 4 Department of Urology, Ajou University Hospital, Ajou University School of Medicine, Suwon, Korea; 5 Department of Urology, Severance Hospital, Yonsei University College of Medicine, Seoul, Korea; 6 Department of Urology, Gangnam Severance Hospital, Yonsei University College of Medicine, Seoul, Korea

**Keywords:** Decision regret, Prostate biopsy, Prostate cancer, Prostate specific antigen, Multicenter study

## Abstract

**Background:**

Many people struggle with the choice in a series of processes, from prostate cancer (PCa) diagnosis to treatment. We investigated the degree of regret after the prostate biopsy (PBx) and relevant factors in patients recommended for biopsy for suspected PCa.

**Methods:**

From 06/2020 to 05/2022, 198 people who performed PBx at three institutions were enrolled and analyzed through a questionnaire before and after biopsy. Before the biopsy, a questionnaire was conducted to evaluate the sociodemographic information, anxiety scale, and health literacy, and after PBx, another questionnaire was conducted to evaluate the decision regret scale. For patients diagnosed as PCa after biopsy, a questionnaire was conducted when additional tests were performed at PCa staging work-up.

**Results:**

190 patients answered the questionnaire before and after PBx. The mean age was 66.2 ± 7.8 years. Overall, 5.5% of men regretted biopsy, but there was no significant difference between groups according to the PCa presence. Multivariate analysis, to identify predictors for regret, revealed that the case when physicians did not properly explain what the prostate-specific antigen (PSA) test was like and what PSA elevation means (OR 20.57, [95% CI 2.45–172.70], *p* = 0.005), low media literacy (OR 10.01, [95% CI 1.09–92.29], *p* = 0.042), and when nobody to rely on (OR 8.49, [95% CI 1.66–43.34], *p* = 0.010) were significantly related.

**Conclusions:**

Overall regret related to PBx was low. Decision regret was more significantly related to media literacy rather than to educational level. For patients with relatively low media literacy and fewer people to rely on in case of serious diseases, more careful attention and counseling on PBx, including a well-informed explanation on PSA test, is helpful.

**Supplementary Information:**

The online version contains supplementary material available at 10.1186/s12889-024-19179-1.

## Introduction

Prostate cancer (PCa) is a common disease in relatively older patients, and some patients often lack an overall medical knowledge of PCa, including a screening process for diagnosis, and an understanding of treatment need [1]. As a result, many people have difficulty making their choice in a series of processes from diagnosis to treatment, and if they experience unrecognizable side effects, they may often resent physicians who treated them and regret deciding on tests and treatments that they have chosen. However, if there were comprehensible explanation of the disease and treatment from the physicians, patients might have fewer regrets even if some problems have occurred in the process of diagnosis or treatment [2]. In line with this background, the importance of so-called shared decision making has been emphasized in various medical fields including Urology [3–5]. Meanwhile, there are several studies reporting treatment-related decision regret in PCa [6–9].

However, it should be noted that these studies are not directly relevant to decision regret for the diagnostic process, including prostate biopsy (PBx). Moreover, there are insufficient studies in Asian countries including South Korea, or Japan compared to some Western studies [10–12] which have reported on this shared decision making in the urology field, especially in the process of diagnosing and treating PCa. This might be a result from combination of factors, including Asian-specific Confucian ideas and the difference between perceptions of doctors, and the tendency that older Asian patients want doctors to take care of themselves by doctors’ decision rather than making their own choices. There are also few large-scaled studies reporting impact of PCa presence on decision regret after PBx.

In this study, as the first step in the process of diagnosing and treating PCa in Asian patients, we investigated the degree of regret after biopsy and various factors related to it for PBx candidate due to suspicion of PCa. We also aimed to evaluate if there is any potential impact of receiving a PCa diagnosis on decision regret concerning PBx.

## Patients and methods

### Study cohort

198 Korean patients who underwent PBx and questionnaires at three tertiary referral centers between 06/2020 and 05/2022 were included in this prospective analysis. Patients over 40 years of age scheduled for PBx who have high serum prostate-specific antigen (PSA) level or who are suspected of PCa from digital rectal examination were included. Patients recommended for biopsy from other institutions were also included. Patients with the poor cognitive ability to the extent that they could not understand the contents of the questionnaire or were deemed inappropriate for study subjects by investigators’ judgment were excluded from the study.

To evaluate the effect of PCa diagnosis on decision regret regarding PBx, patients were classified into two groups based on PCa diagnosis (non-PCa vs. PCa). Irrespective of the institution PBx was conducted, all biopsies were performed with a pre-procedural enema. PBx was performed under ultrasound guidance via a transrectal approach after local anesthesia. Patients were admitted on the day of PBx. Depending on the patients’ condition, they were discharged several hours after PBx or after one more day of observation if desired by the patient or as determined by physicians. All participants provided informed consent. All study protocols were in accordance with the principles of the Helsinki Declaration after approval by institutional review board (IRB number: IS21QIMI0015).

### Acquisition of data

The prospectively maintained clinical database was reviewed. Before the PBx, a questionnaire was conducted to evaluate the sociodemographic information, anxiety scale ( [13], Cronbach’s alpha = 0.71), and health literacy ( [14], Cronbach’s alpha = 0.711). After the PBx, another questionnaire was conducted to evaluate the decision regret scale ( [15], Cronbach’s alpha = 0.81 to 0.92). For patients diagnosed as PCa after biopsy, a questionnaire was conducted when additional tests were performed at PCa staging work-up. Questionnaires were provided as hard copies via one of our investigators to all patients.

For assessment of Anxiety scale, four questions were utilized as follows:

(i) ‘Do you worry about your health?’
(ii) ‘If you have an ache or pain do you worry that it may be caused by a serious illness?’
(iii) ‘Do you find it difficult to keep worries about your health out of your mind?’
(iv) ‘When you read or hear about an illness on TV or radio, does it make you think you may be suffering from that illness?’

For assessment of health literacy, three questions were utilized as follows:

(i) ‘How difficult is it for you to get advice or information about health or medical topics if you need it?’
(ii) ‘How difficult is it for you to understand information that doctors, nurses and other health professionals tell you?’
(iii) ‘You can find written information about health on the Internet, in newspapers and magazines, and in brochures in the doctor’s office and clinic. In general, how difficult is it for you to understand written health information?’

The English version of the questionnaire developed for the study reflects all the detailed information that investigators aimed to analyze (Supplementary file 1).

### Study endpoints

In this study, the primary outcome is to check the degree of regret using the decision regret scale. The secondary outcome is whether there is a significant factor related to regret.

The Decision regret scale questionnaire consisted of five items, each using a 5-point Likert scale with response options including ‘Strongly agree’, ‘Agree’, ‘Neither agree nor disagree’, ‘Disagree’, and ‘Strongly disagree’. To assess the primary outcome, we used the response to question, “I regret the choice that was made,” which, after careful discussion among investigators, was the most direct measure of decision regret out of the five questions. Authors dichotomized the responses to the question (“I regret the choice that was made.“). Then, authors identified the presence of decision regret if the respondent answered ‘Strongly agree’ or ‘Agree’ among five choices, and the absence of decision regret if the respondent answered ‘Neither agree nor disagree’, ‘Disagree’ or ‘Strongly disagree’.

### Statistical analysis

Clinicopathological characteristics were compared between groups (non-PCa vs. PCa) using a Chi-square test for categorical variables and an independent *t*-test or Mann-Whitney U test for continuous variables. The univariate and multivariate logistic regression analyses were performed to detect predictors for decision regret after PBx. Multivariable analysis using variables with a *p*-value < 0.05 in univariate analysis was performed to identify which variables were independently predictive of outcomes [16]. All tests were two sided with a value of 0.05. The statistical analysis was performed using IBM Statistical Package for the Social Science Statistics for Windows (SPSS) version 27.0 (IBM SPSS Statistics, IBM Corp., Armonk, NY, USA).

## Results

190 patients were finally analyzed in the study. 8 patients did not complete the post-biopsy questionnaire due to follow-up loss. The mean age was 66.2 ± 7.8 years. When all patients were classified by PCa presence, there were significant differences between groups in age (years, 64.5 ± 8.0 vs. 68.2 ± 7.2, *p* = 0.001) and serum PSA level (ng/mL, 8.6 ± 6.3 vs. 28.9 ± 86.4, *p* = 0.030). Otherwise, no significant differences were found in biopsy core number, marital status, familial history of PCa, the final level of education, comorbidity, the rate of post-biopsy complication requiring readmission, and the distribution of post-biopsy pain severity between groups (Table 1).

**Table 1:** Demographics and questionnaire key results

Variable	Non-PCa (*n* = 102)	PCa (*n* = 88)	*p* value
Age (year)	64.5 ± 8.0	68.2 ± 7.2	0.001
PrePBx PSA (ng/mL)	8.6 ± 6.3	28.9 ± 86.4	0.030
Number of biopsy core	12.0 ± 0.2	12.0 ± 0.5	0.086
Marital status - Married / Other (%)	93 (91.2) / 9 (8.8)	78 (88.6) / 10 (11.4)	0.745
Highest education level			0.158
Middle / High school / University (%)	21 (20.6) / 42 (41.2) / 39 (38.2)	28 (31.8) / 35 (39.8) / 25 (28.4)	
Familial history of PCa (%)	6 (5.9)	7 (8.0)	0.199
Comorbidity (%)	87 (85.3)	80 (90.9)	0.237
Post-PBx complication requiring readmission (Bleeding or Infection) (%)	0	1 (1.1)	0.280
Nobody to rely on when facing serious illness (%)	3 (2.9)	6 (6.8)	0.011
Low media literacy (%)	46 (45.1)	53 (60.2)	0.037
Health anxiety			0.149
Low / Intermediate / High (%)	32 (31.4) / 59 (57.8) / 11 (10.8)	28 (31.8) / 42 (47.7) / 18 (20.5)	
Sufficient explanation of PSA testing & its elevation			0.083
Yes / No (%)	96 (94.1) / 6 (5.9)	87 (98.9) / 1 (1.1)	
Sufficient explanation of PBx			0.388
Yes / No (%)	99 (97.1) / 3 (2.9)	87 (98.9) / 1 (1.1)	
Post-PBx pain (5-Likert scale)			0.177
Worst pain (%)	20 (19.6)	10 (11.4)	
Very severe pain (%)	44 (43.1)	52 (59.1)	
Moderate pain (%)	8 (7.8)	13 (14.8)	
Mild pain (%)	21 (20.6)	12 (13.6)	
No pain (%)	9 (8.8)	1 (1.1)	

PBx : Prostate biopsy; PCa : Prostate cancer; PSA : Prostate specific antigen

Values are presented as mean ± standard deviation or number (%) against total number

Only one case of PBx related complication was reported. In fact, one patient visited to the emergency department and improved after hospitalization with several days of conservative management including intravenous antibiotic therapy. No one complained of post-PBx erectile dysfunction. There were no statistically significant differences in decision regret based on age. The percentages of regret were 3.6% (1/28) for those < 60 years old, 4.0% (4/100) for those between 60 and 69 years old, and 8.1% (5/62) for those ≥ 70 years old or older (*p* = 0.483).

There were significant differences in some of prebiopsy questionnaire items. As for the question asking about the presence of a person to rely on when facing serious illness, Patients diagnosed as PCa after biopsy significantly responded more to the choice ‘Nobody to rely on when facing serious illness’ than non-PCa patients (6.8% vs 2.9%, *p* = 0.011). Patients diagnosed as PCa after biopsy also significantly answered more to the choice ‘Low media literacy’ compared to non-PCa patients (60.2% vs. 45.1%, *p* = 0.037). There was no difference between the groups in the question asking whether they had sufficient explanation of PSA testing and its elevation (*p* = 0.083) and PBx (*p* = 0.388) (Table 1).

Overall regret rate, our primary outcome, was 5.3% (derived from the item ‘I regret the decision I chose.’). In detail, there was no significant difference between the groups in most items of decision regret scale questionnaire. there was only significant difference between the groups in the answer to the item ‘The choice did me a lot of harm.’ (*p* = 0.014) (Table 2).

**Table 2:** Decision regret scale

Variable	Strongly agree	Agree	Neither agree nor disagree	Disagree	Strongly disagree	*p* value
‘It was the right decision.’						0.157
Non-PCa (%)	23 (23.2)	60 (60.6)	9 (9.1)	3 (3.0)	4 (4.0)	
PCa (%)	14 (16.5)	64 (75.3)	5 (5.9)	2 (2.4)	0 (0)	
‘I regret the choice that was made.’						0.068
Non-PCa (%)	0 (0)	5 (4.9)	15 (14.7)	54 (52.9)	28 (27.5)	
PCa (%)	0 (0)	5 (5.7)	7 (8.0)	62 (70.5)	14 (15.9)	
‘I would go for the same choice if I had to do it over again.’						0.146
Non-PCa (%)	17 (16.8)	62 (61.4)	8 (7.9)	9 (8.9)	5 (5.0)	
PCa (%)	10 (11.5)	65 (74.7)	5 (5.7)	7 (8.0)	0 (0)	
‘The choice did me a lot of harm.’						0.014
Non-Pca (%)	0 (0)	7 (7.3)	10 (10.4)	47 (49.0)	32 (33.3)	
PCa (%)	0 (0)	6 (7.1)	12 (14.1)	56 (65.9)	11 (12.9)	
‘The decision was a wise.’						0.170
Non-PCa (%)	23 (22.8)	56 (55.4)	15 (14.9)	5 (5.0)	2 (2.0)	
PCa (%)	16 (18.8)	60 (70.6)	6 (7.1)	3 (3.5)	0 (0)	

PCa : Prostate cancer patient group; Non-PCa : Non-prostate cancer patient group

Multivariable logistic analysis revealed that decision regret after PBx was significantly associated when there is no one to rely on in the event of a serious illness (odds ratio [OR]: 8.49, 95% confidence interval [CI]: 1.66–43.34, *p* = 0.010), when patients thought they had low media literacy (OR: 10.01, 95% CI: 1.09–92.29, *p* = 0.042), and when they thought they had insufficient explanation of PSA testing and its elevation (OR: 20.57, 95% CI: 2.45–172.70, *p* = 0.005) (Table 3).

**Table 3:** Factors associated with regret after prostate biopsy

			Univariable analysis			Multivariable analysis		
			OR	CI	p value	OR	CI	p value
Age			1.04	0.95–1.13	0.385			
Prebiopsy PSA			1.01	0.99–1.01	0.080			
No one to rely on in the event of a serious illness			8.14	1.80-36.84	0.006	8.49	1.66–43.34	0.010
Low media literacy			9.00	1.12–72.51	0.039	10.01	1.09–92.29	0.042
Health anxiety		Low	Ref		0.419			
		Intermediate	0.78	0.17–3.63	0.755			
		High	2.19	0.41–11.60	0.356			
Highest education level		University	Ref		0.224			
		High school	3.52	0.65–18.99				
		Middle school	1.26	0.20–7.76				
PCa diagnosis			1.17	0.33–4.18	0.810			
Insufficient explanation of PSA testing and its elevation			2.50	1.26–4.97	0.009	20.57	2.45–172.70	0.005
Well explanation of prostate biopsy			0.15	0.01–1.62	0.118			

PCa : Prostate cancer; PSA : Prostate specific antigen

## Discussions

Decision regret is one of the issues that should always be kept in mind for patients who are scheduled for PBx. Substantial proportion of patients diagnosed as PCa after biopsy regret their initial treatment decision [17, 18]. Davison et al [17] reported 4% of respondents expressed regret over their decision to have surgery. Lin’s study [18] reported 31% of the participants reported experiencing regret after receiving an radical prostatectomy. Wallis et al. conducted a prospective population-based study from 5 population-based Surveillance, Epidemiology, and End Results registries across the US. They assessed regret at 5 and 3 years after treatment by using a validated prostate cancer–oriented scale among 2,072 participants who underwent radical prostatectomy, radiotherapy, or active surveillance. Of these, 279 men reported having treatment-related regret at 5 years [6].

Factors associated with treatment regret among men with PCa affect quality of life and therapeutic relationships among patient, caregiver, and clinician. One study [19] reported time pressure, erectile dysfunction, and satisfaction with sexual life predict decisional regret in men with localized PCa. Lavery et al [20] revealed that superior erectile function and baseline continence independently predicted increased postoperative decision regret. Moreover, older age, postoperative incontinence, postoperative erectile dysfunction and longer time from operation were significant predictors of increased decision regret. Mahal et al [21] evaluated the impact of race on treatment regret among men with biochemically recurrent PCa after surgery or radiation. Among those without sexual problems, African American men had more treatment regret than non-African American men (26.7% vs. 8.4%), whereas among those with sexual problems, there was no difference in treatment regret between African American and non-African American men (18.8% vs. 17.3%). Another study [22] concluded spirituality may aid patients diagnosed as PCa after biopsy experience less decisional regret and stronger spiritual beliefs were associated with less decision regret. Recent study [23] also reported financial burden was associated with treatment regret at 3 years after treatment for localized PCa.

There were some studies supporting our findings. One population-based study reported educational length was associated with the chance of having a PBx in men with PSA 4–10 ng/ml. It also revealed the time between the PSA test and the biopsy was longer in men with a short education [24]. This finding suggests that accurate and understandable explanations on PSA testing and its elevation are important for patients having potential for decision regret after biopsy. Another study [12] investigated the association between health literacy, numeracy, prostate-related knowledge and treatment regret. It identified lower prostate-related knowledge in those with poor health literacy. Patients with regret were more likely to be not married, and score lower on the literacy and numeracy scales. These results might be someway related to our findings of ‘relationship among decision regret after biopsy, mass literacy, and presence of caregiver or tower of strength’.

The strength of this study was that this study focuses on the process of prostate cancer diagnosis and analyzes decision regret related to PBx, which is the core process. In fact, most of the studies done so far have dealt with decision regret according to the choice of treatment modality or post-treatment follow-up after diagnosis of prostate cancer. Moreover, there are few studies like this in the Asian cohort, and this leading study will serve as a cornerstone for subsequent studies in the near future, which is another strength of this study. In addition, this is the first study to analyze a multicenter cohort and find that decision regret is more significantly related to media literacy than to educational attainment. While higher levels of education might be often correlated with higher levels of media literacy, it’s important to note that correlation does not necessarily imply causation, and there may be exceptions like our results. It can also be deduced from our results that while an educational degree can provide a foundation for media literacy, it’s not critical determinant of decision regret from PBx. We also found that being diagnosed with PCa had no significant impact on decision regret after PBx. Our results might help minimize decision regret, as it could be conferred that it is the background characteristics of the men undergoing PBx, rather than the biopsy results themselves, that affect decision regret related to PBx. Nevertheless, present study is not devoid of limitations. First, even with cohort from three tertiary centers, the study population was still small due to lack of voluntary participation by patients. It is worth noting that the results of our multivariable analysis might not be readily applicable to the real clinical setting due to the limited number of patients who expressed decision regret after PBx. The wide CIs for the OR, despite significant *p*-values, make it challenging to draw definitive conclusions. Authors acknowledged that only 10 patients reported decision regret, and it might be difficult to make meaningful comparisons between these 10 patients and the 180 patients who did not report decision regret. Second, we could not fully eliminate a selection and recall bias. Moreover, it was a study from a single ethnicity. Our study also posed a challenge in assessing whether the expense and hospitalization influenced the decision regret after PBx. In our multicenter study, there might be little variation both in the cost of prostate biopsy across institutions, and in the cost of biopsy procedure costs with or without hospitalization because costs are mostly reimbursed by domestic national health insurance. Nevertheless, the authors were unable to examine these variations specifically. Finally, linguistic validation of the Korean version of the above three questionnaires [13–15] has not been performed, but all of them are simple and clear questionnaires, and the number of questions is relatively small in short form, so it is considered that Korean validation is not absolutely necessary. Though the respondents in the study did not have any difficulty completing the questionnaire, it is suggested that future studies use a linguistically validated questionnaire to ensure the reproducibility of our results. Further prospective studies need to overcome these limitations.

## Conclusions

Overall regret related to PBx was low. Decision regret was more significantly related to media literacy rather than to educational level. For patients with relatively low literacy of media and fewer people to rely on due to serious diseases, more careful attention and counseling on PBx, including a well-informed explanation of the PSA test, is helpful. Further prospective randomized controlled trials are mandatory to identify predictors of decision regret. Such information will benefit patients and physicians to ease shared decision-making, improve patient’s quality of life, and minimize their medical regret.

### Electronic supplementary material

Below is the link to the electronic supplementary material.

Supplementary Material 1

## Data Availability

The datasets used and/or analyzed during the current study available from the corresponding author on reasonable request.
