# Supplementary material for: Decision regret after prostate biopsy for prostate cancer diagnosis: a Korean multicenter cohort study
Source: BMC Public Health. 2024 Jun 28;24:1725. doi: 10.1186/s12889-024-19179-1 (PMC11212167; doi:10.1186/s12889-024-19179-1)
Supplement: Supplementary file 1 — Supplementary Material 1 [file 12889_2024_19179_MOESM1_ESM.pdf]

<Survey Form> (1)

You are scheduled to have a prostate biopsy. This questionnaire is designed to obtain some basic information about patients who are about to undergo a biopsy. You can rest assured that your answers will be kept strictly confidential.

(1) Fill in the date you are filling out the questionnaire. Year ____Month __Day __

(2) Can someone help you fill out this questionnaire? Yes □ No □

(3) If you have someone helping you fill out the questionnaire, please list their relationship to the patient. (Example; wife, children...) ____

(4) This question asks if you are married.

Marital status □ Never been married □ Widowed □ Separated □

(5) Do you have a family member currently living with you? Yes □ No □

(6) Your highest level of education

Middle school or lower □ High school □ College or higher □

(7) Do you currently have a job? Yes □ No □

(8) Has your father or brother been diagnosed with prostate cancer?

Yes □ No □ Don't know □

(9) Is this your first prostate biopsy? Yes □ No □

If not, please indicate how many prostate biopsies you have had. _______

(10) Are you concerned about your health?

Never/rarely □ Sometimes □ Often □ Many times □ A lot of the time □

(11) If you feel an ache or pain somewhere, do you think it could be caused by a serious illness?

Never/rarely □ Sometimes □ Often □ Many times □ A lot of the time □

(12) Do you find it hard to shake the thought that you might be sick somewhere?

Never/rarely □ Sometimes □ Often □ Many times □ A lot of the time □

(13) Do you often find yourself thinking that you could be sick when you hear about various diseases and conditions on television, radio, or other media?

Never/rarely □ Sometimes □ Often □ Many times □ A lot of the time □

(14) How many people do you think you could count on if you had a serious illness?

None □ 1-2 people □ 3-4 people □ 5 or more people □

(15) Were you healthy before you had your prostate biopsy?

I was very healthy □ Healthy □ Fairly healthy □ Not very healthy □ Very poorly healthy □

(16) Please check the conditions you had prior to your prostate biopsy. (Duplicates may be marked)

Heart disease □ Lung disease □ Stroke □ Diabetes □ Depression □
High blood pressure □ Chronic colorectal disease □ Cancer
□ Other ____________________(Write your own)

(17) I find it very easy to get information about my health when I need it.

0 (very difficult) □ 1 (a little difficult) □ 2 (a little easy) □ 3 (very easy) □

(18) It is very easy for me to understand when I hear a doctor or nurse's explanation.

0 (very difficult) □ 1 (a little difficult) □ 2 (a little easy) □ 3 (very easy) □

(19) I find it very easy to understand when I read media on the internet, brochures, etc.

0 (very difficult) □ 1 (a little difficult) □ 2 (a little easy) □ 3 (very easy) □

Your survey is complete, thank you for taking the time to complete it.

< Survey Form> (2)

You had a prostate biopsy a few weeks ago, and this questionnaire is designed to learn about your experiences and feelings about the process. You can rest assured that your answers will be kept strictly confidential.

(1) As for prostate specific antigen (PSA) test performed before biopsy, Have you been told by your doctor what kind of test it is for the PSA test and how it will proceed after the abnormal range comes out?

□ Yes, it was explained to me and I understood it well.
□ Yes, I was told, but I need more explanation.
□ No, I did not receive any explanation, but I don't think I need an explanation and I think the doctor will do a good job.
□ No, I did not receive any explanation. I would have liked the doctor to explain things in more detail.

(2) Have you been explained and guided to ensure you are fully prepared before the biopsy? (e.g., biopsy process, precautions, hematuria or pain that may occur after the biopsy and precautions to avoid them, etc.)

□ Yes, it was explained to me and I understood it well.
□ Yes, I was told, but I need more explanation.
□ No, I did not receive any explanation, but I don't think I need an explanation and I think the doctor will do a good job.
□ No, I did not receive any explanation. I would have liked the doctor to explain things in more detail.

(3) Did a doctor or nurse explain that you might get sick during a biopsy?

□ Yes, I was preparing because I knew it could hurt during the examination.
□ Yes, but I was not prepared for the pain I felt during the examination.
□ Yes, but the pain wasn't as bad as I thought.
□ I didn't hear any explanation.

(4) The following is what you feel after prostate biopsy. Please record how much you felt.

	Strongly agree	Agree	Neither agree nor disagree	Disagree	Strongly disagree
1. I was relieved after the biopsy.					
2. I was embarrassed.					
3. Biopsy was necessary.					
4. I felt pain.					
5. It was no big deal.					
6. I was anxious.					
7. I was undignified.					
8. It stressed me out.					
9. I was scared.					
10. After the biopsy, I felt at ease.					
11. It was unpleasant.					
12. It did not take much time than I thought.					
13. I am very tired.					
14. It was not as bad as I thought.					
15. It was uncomfortable.					
16. I was worried.					
17. I was glad I did biopsy.					
18. It was the right decision.					
19. I regret the choice that was made.					
20. I would go for the same choice if I had to do it over again.					
21. The choice did me a lot of harm.					
22. The decision was a wise.					

Your survey is complete, thank you for taking the time to complete it.
